# Scrotal Extramammary Paget’s Disease in an Elderly Caucasian Male

**DOI:** 10.7759/cureus.29486

**Published:** 2022-09-23

**Authors:** Jaimie M Barrera, Tharangini Vummadi, Victor J Mihal

**Affiliations:** 1 Medicine, Edward Via College of Osteopathic Medicine, Blacksburg, USA; 2 Family Medicine, Sentara Halifax Family Medicine, South Boston, USA

**Keywords:** mohs micrographic surgery, dermatological malignancy, genital lesion, scrotal rash, extramammary paget’s disease

## Abstract

Extramammary Paget’s disease (EMPD) is a rare dermatologic malignancy affecting regions with a dense population of apocrine glands within the intraepithelial tissue, including the vulva, perineum, axilla, scrotum, and penile regions. Clinical presentation varies from being asymptomatic to burning, painful, and pruritic lesions. As a result, it could be misdiagnosed for other dermatologic diseases. Our case report discloses a patient with an erythematous pruritic lesion that was initially treated with topical antifungal therapy. After failed treatment, a biopsy revealed EMPD of the scrotum. With no standard guidelines in the treatment of EMPD, there are different treatment modalities for the disease. Mohs micrographic surgery currently is the preferred treatment modality presenting with the lowest rates of recurrence. With early diagnosis and treatment, the five-year survival rate for patients with primary EMPD is 87%. Therefore, there should be a high level of clinical suspicion for EMPD in patients presenting with pruritic lesions in areas with apocrine glands that have failed initial medical treatment.

## Introduction

Extramammary Paget’s disease (EMPD) is a rare dermatologic malignancy in regions with abundant apocrine glands within the intraepithelial tissue. The area most affected is the vulva followed by the perineum, axilla, scrotum, and penile regions [1]. With a few hundred cases reported worldwide, an accurate incidence rate of EMPD is unknown, however, it commonly affects the older population between 60 and 80 years of age [1,2]. The prevalence of EMPD interestingly varies regionally among racial and gender groups. The prevalence of EMPD in Europe is approximately 0.7 per 1 million, and in Asia it is 0.4 per 1 million. Although the disease affects both males and females, it is rare in males [2]. Among the Caucasian population, EMPD is more prevalent among women compared to men [2]. Clinically, patients initially present with a lesion reporting nonspecific symptoms such as erythema, pruritus, burning, pain, and scaly plaque-like rash.

## Case presentation

**Figure 1:**
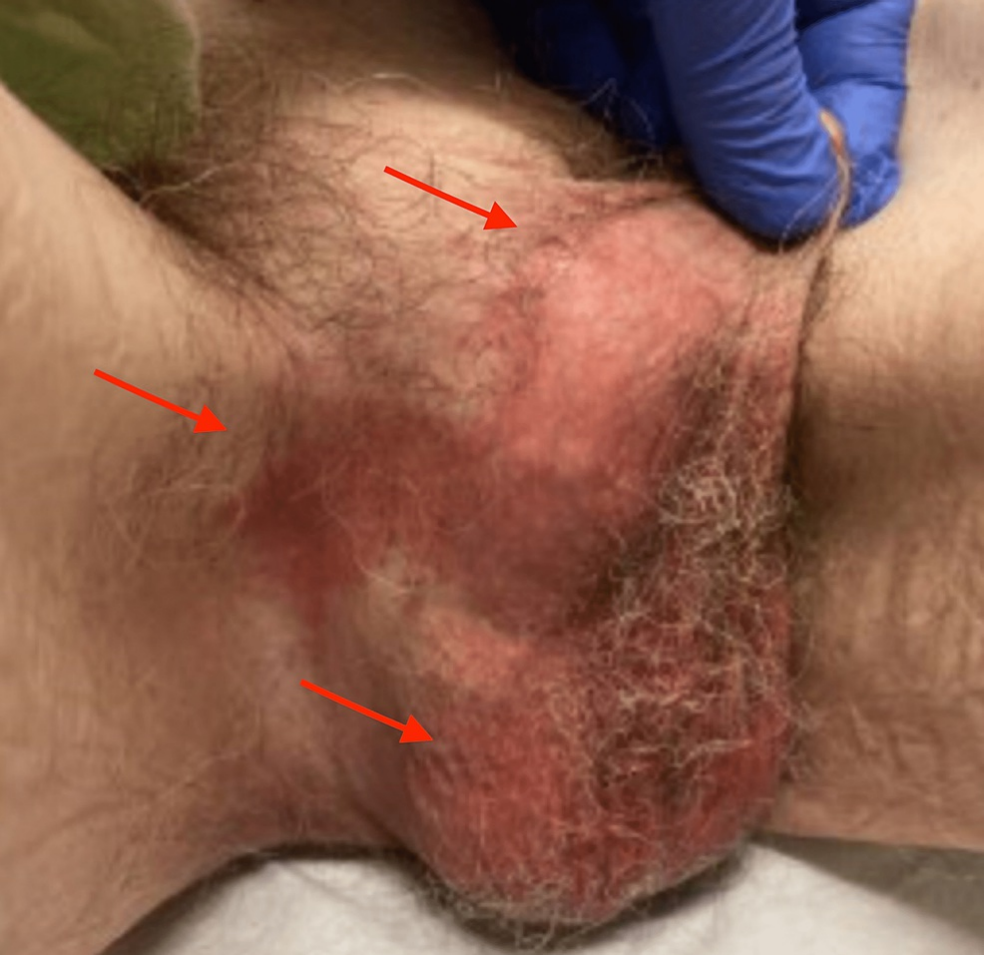
The rash at the time of biopsy exhibiting erythema and extension from scrotum towards the penis and inguinal region.

A 77-year-old Caucasian male with a history of congenital phimosis, prostate cancer in remission now for 13 years status post brachytherapy, hypertension, and gastroesophageal reflux disease presented to the primary care clinic with a complaint of persistent itching, redness, and discomfort in the scrotal area for over five months. The patient had tried clindamycin cream, nystatin, and topical steroids with no relief. He was seen four months later at the same clinic presenting with similar complaints in addition to bleeding. He was then diagnosed with tinea cruris and prescribed clotrimazole 1% and betamethasone dipropionate 0.5% cream which helped resolve his symptoms. Examination revealed an erythematous scrotum with a patch of erythema extending into the inguinal region (Figure 1). Based on the physical appearance and similar presentation in the past, a second trial of clotrimazole 1% and betamethasone dipropionate 0.5% cream treatment was prescribed. The patient returned to the clinic six weeks later with unresolved symptoms.

**Figure 2:**
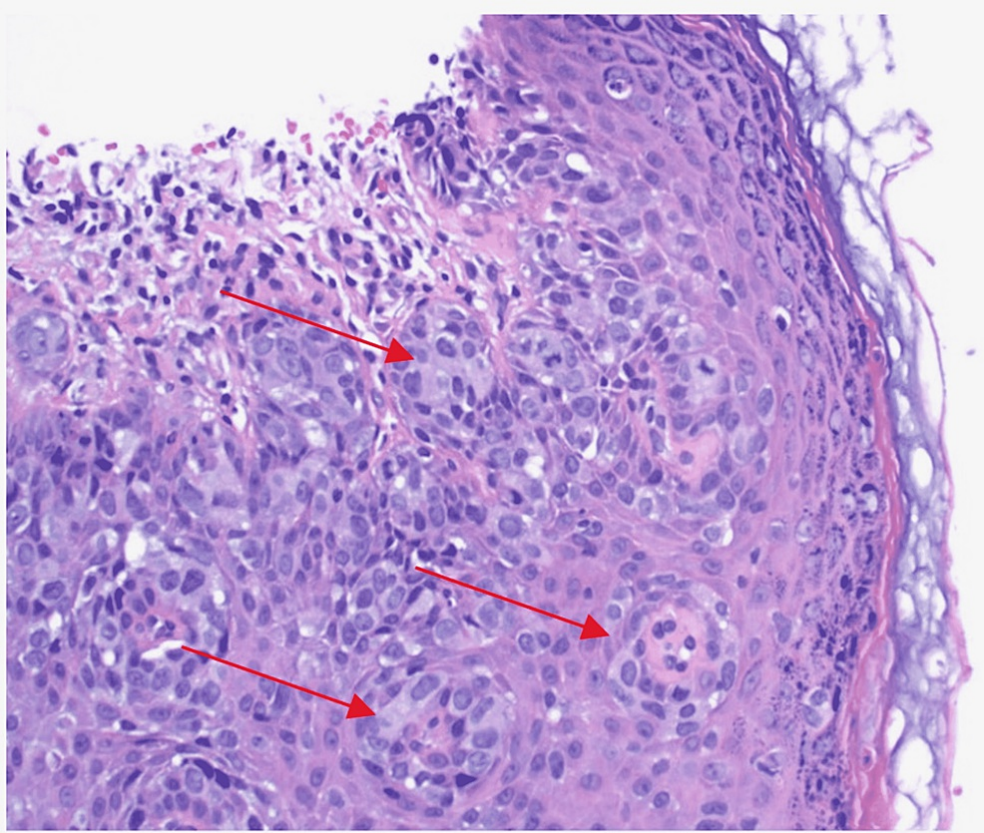
Punch biopsy of the scrotal lesion, showing classic pagetoid cells with pale, vacuolated cytoplasm arranged in clusters above the basal layer of the epidermis. Red arrows: clustered pagetoid cells.

**Figure 3:**
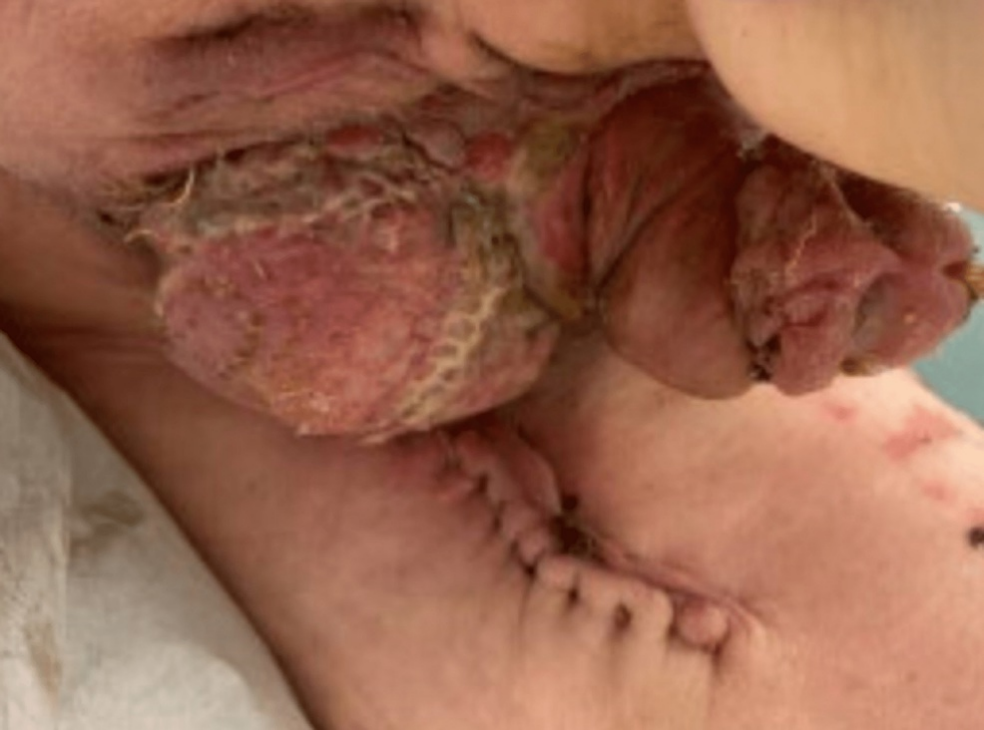
The affected area after repair with split-thickness autograft.

Due to clinical suspicion of malignancy, a punch biopsy of the scrotum was obtained. Histopathological examination of the biopsy sample showed clusters of large cells with pale, vacuolated cytoplasm just above the basal layer of the epidermis (Figure 2). The tumor cells showed atypia, however, there was no invasion into the dermis. Immunohistochemically, the intraepidermal neoplastic cells were positive for GATA3 and cytokeratin 7 and negative for S100 and all other markers including prostate-specific and sensitive marker Nkx3.1. The morphological feature in addition to the immunohistological findings was diagnostic of EMPD. Mohs micrographic surgery (MMS) was performed by urology with extensions of sectioning until negative margins were obtained. Due to an extensive area of defect, including the entire scrotum, about 50% of the perineum from the left to the right thigh crease, one-third of the penile shaft at the base and 5 x 5 cm of the left thigh, a split-thickness autograft was used to reconstruct the area (Figure 3). The postoperative plan for the patient was to apply topical over-the-counter Eucerin cream twice daily to donor and recipient sites. The patient was instructed to follow-up with his urologist and primary care physician in three months to monitor for any recurring signs of the malignancy or sooner if there are any signs of complications, such as bleeding, itching, fever, redness, or discoloration of graft skin.

## Discussion

EMPD is an uncommon neoplastic skin condition most prevalent among the elderly and female populations and is extremely rare among males. The disease primarily affects areas with apocrine sweat glands, including the vulva, perianal, axilla, scrotum, and penile regions [3].

Clinical presentation can range from asymptomatic lesions to nonspecific erythema, pruritus, and pain. Pruritus, reported in approximately 70% of cases, is the most common symptom; approximately 10% of cases are asymptomatic [4]. The appearance differs based on the site of the lesion, progression of disease, and stage at presentation [5]. Lesions can vary from being well-circumscribed, hypopigmented, or dry plaques to having irregular borders, hyperpigmented, or cobblestone-like papules [5,6]. Therefore, the disease can often be misidentified as other benign dermatological lesions. The most common misdiagnoses are inflammatory fungal infections, contact dermatitis, seborrheic eczema, psoriasis, lichen sclerosus, lichen planus, and intertrigo [3]. Multiple topical treatments are tried often leading to a delay in definitive diagnosis and treatment that contribute to disease progression and poor prognosis [7]. Therefore, EMPD should be considered when a patient presents with a persistent pruritic lesion and failed initial medical therapy [7].

A biopsy with immunohistological staining is the preferred method to diagnose and determine the cell type of origin [5-7]. The most common staining markers used are the following: cytokeratin 7 (CK7), CK20, gross cystic disease fluid protein 15 (GCDFP-15), Human Melanoma Black 45 (HMB45), anti-cytokeratin (CAM 5.2), c-erb, S100 protein, and epithelial membrane antigen (EMA) [7]. If immunohistological staining presents with positive biomarkers, as in this case, additional testing should be conducted to identify further underlying malignancies, such as squamous cell carcinoma, basal cell carcinoma, prostate cancer, melanoma, and breast cancer [7]. Histological spread is not atypical and therefore MMS is considered appropriate therapy to reduce risk of recurrence [8,9]. Imaging with ultrasonography or computed tomography is highly suggested for large invasive lesions or lesions with underlying malignancy. Examining for lymphadenopathy is also recommended for further biopsy [10].

Due to the low prevalence of the disease and limited scientific evidence, there are no standard guidelines for the treatment of EMPD [6]. Treatment modalities for EMPD depend on the size of the lesion, advancement of disease, lymph node spread, bone metastasis, and the patient’s health status and preferences [11,12]. Previously, standard treatment was a wide local excision procedure but resulted in recurrence rate ranging from 20% to 60% [9,12]. Most recently, MMS has become the most common and preferred treatment for EMPD [12]. MMS uses intraoperative frozen technique to guide resectioning of the lesion and removal of the lesion in its entirety. Therefore, there is a lower recurrence rate of 8-26% with MMS therapy in comparison to wide local excision [12]. A complete resection of large lesions might not always be possible. In case of extensive lesions, a clearance of the immediate tumor area followed by adjuvant therapy with topical immunomodulators, or radiation therapy and close observation is recommended [10]. With complete surgical resectioning being the treatment of choice, areas of defect tend to be extensive, which often cannot be repaired through primary closure [7]. As a result, most cases require split-thickness skin grafting or local/regional flap reconstruction [7].

Although most cases of EMPD are localized to the epidermis, there is potential for dermal invasion and metastasis resulting in poor prognosis [7,13]. The five-year survival rate for patients with primary EMPD is 87% [13]. Patients with localized disease have a five-year survival rate of 92% and lower rates in patients with regional metastases and distant metastases at 77% and 16%, respectively [13]. Due to low rates of occurrence of this disease and even fewer research studies relating to EMPD, long-term follow up and frequent monitoring would be indicated.

## Conclusions

Given the varying degrees of presentation and the rarity of the disease, the diagnosis of EMPD can be mistaken for other common dermatological conditions. As a result, affected patients often fail to receive initial proper treatment. Therefore, there should be a high level of clinical suspicion for EMPD in patients presenting with pruritic lesions in areas with high apocrine glands, specifically the vulva, perineum, scrotum, and penile regions. A biopsy should be performed for patients who do not respond to the initial standard of care. Surgical intervention with MMS is the mainstay of treatment. Early diagnosis and treatment alone are associated with reduced recurrence and increased survival rate.
